# Understanding Canadian stakeholders’ views on measuring and valuing health for children and adolescents: a qualitative study

**DOI:** 10.1007/s11136-024-03618-y

**Published:** 2024-03-05

**Authors:** Feng Xie, Shitong Xie, Eleanor Pullenayegum, Arto Ohinmaa

**Affiliations:** 1 Department of Health Research Methods, Evidence, and Impact, McMaster University, Hamilton, Canada; 2 School of Pharmaceutical Science and Technology, Tianjin University, Tianjin, China; 3 The Hospital for Sick Children, Toronto, Canada; 4 Dalla Lana School of Public Health, The University of Toronto, Toronto, Canada; 5 School of Public Health, University of Alberta, Edmonton, Canada; 6 Centre for Health Economics and Policy Analysis, McMaster University, Hamilton, Canada

**Keywords:** Health state preference, EQ-5D-Y, Child health valuation, Stakeholder engagement, Qualitative

## Abstract

**Objective:**

Valuing child health is critical to assessing the value of healthcare interventions for children. However, there remain important methodological and normative issues. This qualitative study aimed to understand the views of Canadian stakeholders on these issues.

**Methods:**

Stakeholders from health technology assessment (HTA) agencies, pharmaceutical industry representatives, healthcare providers, and academic researchers/scholars were invited to attend an online interview. Semi-structured interviews were designed to focus on: (1) comparing the 3-level and 5-level versions of the EQ-5D-Y; (2) source of preferences for valuation (adults vs. children); (3) perspective of valuation tasks; and (4) methods for valuation (discrete choice experiment [DCE] and its variants versus time trade-off [TTO]). Participants were probed to consider HTA guidelines, cognitive capacity, and potential ethical concerns. All interviews were recorded and transcribed verbatim. Framework analysis with the incidence density method was used to analyze the data.

**Results:**

Fifteen interviews were conducted between May and September 2022. 66.7% (*N* = 10) of participants had experience with economic evaluations, and 86.7% (*N* = 13) were parents. Eleven participants preferred the EQ-5D-Y-5L. 12 participants suggested that adolescents should be directly involved in child health valuation from their own perspective. The participants were split on the ethical concerns. Eight participants did not think that there was ethical concern. 11 participants preferred DCE to TTO. Among the DCE variants, 6 participants preferred the DCE with duration to the DCE with death.

**Conclusions:**

Most Canadian stakeholders supported eliciting the preferences of adolescents directly from their own perspective for child health valuation. DCE was preferred if adolescents are directly involved.

**Supplementary Information:**

The online version contains supplementary material available at 10.1007/s11136-024-03618-y.

## Introduction

There has been a growing interest in measuring and valuing health for children and adolescents in recent years [1]. Since the development of the EQ-5D-Y, a child-friendly version of the EQ-5D in 2010 [2, 3], a series of methodological research and empirical applications using the EQ-5D-Y have been conducted worldwide [4–13]. For adults, methods for the measurement and valuation of health have been well established. However, there are unique challenges when measuring and valuing child health. It has been demonstrated that health state preferences of children and adolescents differ from those of adults and therefore led to the argument supporting the development of a separate value set for children and adolescents [4, 5, 14]. Further research was then conducted to explore the impact of the perspective used in the valuation tasks [8, 15]. In the meantime, the EQ-5D-Y has been applied in observational research and routine outcome assessments [11–13].

In 2020, an international valuation protocol for the 3-level of the EQ-5D-Y (EQ-5D-Y-3L) was published [16]. A few countries have developed a value set for the instrument using the protocol [17–24]. In the meantime, the EQ-5D-Y-5L, a revised version of EQ-5D-Y with expanded response levels, has been developed to measure health for children and adolescents with improved sensitivity [25]. Despite the publication of the valuation protocol, key issues remain, including the choice between the EQ-5D-Y-3L and Y-5L (the difference between these two health state descriptive systems and the acceptability of increasing response levels), source of health state preferences (who should be asked to provide health state preferences for child health valuation?), and perspective (adult’s or child’s own perspective?) [13, 26].

The choice of valuation methods used to elicit preferences are also critical for valuing health for children and adolescents. Traditional methods such as time trade-off (TTO) have been established in adult populations. However, empirical evidence about these methods in child health valuation is lacking. Discrete choice experiment (DCE) is potentially promising with its lower cognitive burden and the feasibility through online survey. The latent utility generated by DCE needs to be anchored using additional tasks, for example, with immediate death presented as an additional option or duration presented as an additional attribute [27–29]. Whether these variations of DCE work for child health valuation is less clear [13, 26].

Besides, child health valuation study designed according to such a protocol would fail to incorporate considerations deemed important in local contexts. A utility value set will eventually be used to support health technology assessment (HTA) and local reimbursement decision making in which the priority is to efficiently allocate resources in the local setting rather than to make an international comparison. Engagement of stakeholders is therefore recommended to identify and incorporate context-specific factors into child health valuation [30]. While there is a lacking such evidence of taking views of stakeholders into account for valuing health for children and adolescents.

Research in child health has been a top priority in Canadian health research. A prominent example is the Ontario Child Health Study (OCHS) that has been conducted since 1983 [31, 32]. Canadian researchers developed one of the first child health instruments, the Health Utility Index (HUI) in 1992 [33]. Although no specific instrument is recommended by the HTA agency in Canada, most of the research related to child health valuation has been limited to HUIs. To date only a few studies have used the EQ-5D-Y to measure child health in Canada [6, 34, 35]. The aim of this study was, therefore, to understand the views of Canadians stakeholders on the issues related to measuring and valuing child health using the EQ-5D-Y, including the preference of using the EQ-5D-Y-3L or the Y-5L, the source of health state preferences, the perspective of the valuation task, the preference of using TTO or DCE approaches, and potential ethical issues when directly asking children to answer the valuation tasks.

## Methods

### Overview

Semi-structured qualitative interviews were conducted with Canadian stakeholders between May and September 2022. This study has been reviewed and approved by the Hamilton Integrated Research Ethics Board (HIREB) (Project no. 14550). Informed consent was obtained from all study participants before the interview.

### Stakeholders

Stakeholders in Canada were identified from authors’ networks. We recruited participants from the sectors that were determined to be relevant and represent broad coverage of expertise and background for the purpose of the interviews: (1) HTA agencies; (2) healthcare providers for pediatric populations; (3) academia; and (4) the pharmaceutical industry. The sample size was predetermined at 15 to ensure the information saturation as well as a minimum of 2 participants in each sector. Participants were offered a gift card of 50 Canadian dollars as a token of appreciation for their time and effort.

### Online interview

A one-hour interview was conducted with each participant on Zoom. All interviews were led by one author (FX) using a pre-developed script. Each interview was comprised of three parts: 1) demographic questions (i.e., sex, age, education, employment, parental status), and questions about prior experience in health economic evaluation and health utility measurement; 2) introduction on the HTA process and the role of preference-based instruments in supporting coverage decision policy making in Canada; and 3) child health measure and valuation.

During the third part of the interview, both 3-level and 5-level of the EQ-5D-Y were presented to the participants with the differences in the descriptive system highlighted. The participants were asked to indicate which instrument they think is more appropriate to measure child health and why. Then the process and the methods commonly used in child health valuation (i.e., TTO and DCE) were described. The first question was who should be asked to provide their health state preferences for child health valuation (i.e., source of health state preferences). To understand the rationale for their choice, we used a series of probing questions related to the HTA guidelines on health valuation, cognitive capability, and ethical considerations. The participants were then asked about which perspective should be used to frame the valuation tasks. Lastly, the participants were asked to choose which preference elicitation methods should be used in child health valuation. For participants who preferred the use of DCE, a further question about the preference of two variants of DCE (with duration or compared with immediate death) for anchoring the latent utilities. At the end of the interview, the participants were given an opportunity to raise any questions.

### Data analysis

All interviews were recorded. The characteristics of participants were summarized. The audio recordings were transcribed verbatim by one author (SX) and assessed for quality by another author (FX). All the transcripts were then analyzed using the framework analysis [36] in six steps [37]:

(1) *Familiarization* One author (SX) read and re-read the transcripts, while listening to the audio recordings, to obtain familiarity with the data. (2) *Coding* One author (SX) assigned codes (i.e., summary labels) to the transcripts. Coding was informed by the key issues mentioned above and emerging contents from the transcripts. (3) *Developing the framework* Two authors (FX and SX) discussed coding and consensus was reached on a provisional analytic framework featuring a set of codes that were grouped into themes. A draft framework was then applied to the remaining transcripts, while noting any new codes that emerged. The framework was finalized when all transcripts were coded. (4) *Indexing* the framework was used to systematically index the transcripts. (5) *Charting* Microsoft Excel was used to summarize the indexed data in a matrix, with one row per code and one column per participant. Each cell in the matrix was then populated using verbatim data from the transcripts. (6) *Interpretation* All authors agreed on the final interpretation of the data, including descriptive summary for each of the themes, codes contained within each theme, and supporting quotations of the verbatim data from the transcripts. Verbatim descriptions quoted in this paper are labelled for individual stakeholders (A for academia, H for healthcare provider, G for government/HTA agency, and I for industry). In addition, the difference of results between the participants who had previous experience of utility measurement and those who had not were compared.

## Results

We approached 17 stakeholders and 15 agreed to participate. The mean (SD) time duration of the interview was 51.3 (9.5) mins. As shown in Table 1, 11 (73.3%) participants were female. Five (33.3%) participants were aged 31–40 years, three (20.0%) 41–50 years, and seven (46.7%) 51–65 years. Ten (66.7%) participants had PhD degree and 13 (86.7%) were parents. Two (13.3%) participants were from the pharmaceutical industry, four (26.7%) the HTA agencies, four (26.7%) health care providers, and five (33.3%) universities (including two child health researchers and a bioethicist). Most participants had previous experiences of economic evaluation (*n* = 10, 66.7%), were familiar with the concept of health utility (*n* = 13, 86.7%) or involved in measuring health utilities (*n* = 9, 60.0%). Table 2 provides a summary of responses to the key interview questions.

**Table 1:** Characteristics of participants in the stakeholder engagement

	*N*	%
*Sex*		
Female	11	73.3
Male	4	26.7
*Age group*		
31–40	5	33.3
41–50	3	20.0
51–65	7	46.7
*Degree*		
PhD	10	66.7
MD	2	13.3
Master	2	13.3
Bachelor	1	6.7
*Parental status*		
Yes	13	86.7
No	2	13.3
*Current employer*		
Academia	5	33.3
Healthcare provider	4	26.7
Government/HTA agency	4	26.7
Industry	2	13.3
*Experience of economic evaluation*		
Yes	10	66.7
No	5	33.3
*Familiarity with health utility*		
Yes	13	86.7
No	2	13.3
*Experience of health utility measurement*		
Yes	9	60.0
No	6	40.0

**Table 2:** Summary of responses to the interview questions

	Total sample (*N* = 15)	Participant with experience of utility measurement (*N* = 9)	Participant without experience of utility measurement (*N* = 6)
*EQ-5D-Y-3L versus EQ-5D-Y-5L?*			
EQ-5D-Y-5L	11 (73.3%)	7 (77.8%)	4 (66.7%)
EQ-5D-Y-3L	3 (20.0%)	1 (11.1%)	2 (33.3%)
No strong preference	1 (6.7%)	1 (11.1%)	0 (0%)
*Who should complete the valuation tasks?*			
Children and adolescents	12 (80.0%)	7 (77.8%)	5 (83.3%)
Adults	2 (13.3%)	2 (22.2%)	0 (0%)
Mix of both	1 (6.7%)	0 (0%)	1 (16.7%)
*How to frame the perspective of the tasks?*			
Children and adolescents thinking themselves	12 (80.0%)	7 (77.8%)	5 (83.3%)
Own child’s friend group or peer group	1 (6.7%)	0 (0%)	1 (16.7%)
General child without specification	1 (6.7%)	1 (11.1%)	0
No strong preference	1 (6.7%)	1 (11.1%)	0
*Time trade-off (TTO) versus Discrete choice experiment (DCE)?*			
DCE	11 (73.3%)	5 (55.6%)	6 (100%)
TTO	2 (13.3%)	2 (22.2%)	0
No strong preference	2 (13.3%)	2 (22.2%)	0
DCE with death option versus DCE with duration dimension for anchoring? *			
DCE with duration	6 (54.5%)	3 (60.0%)	3 (50%)
DCE with death	2 (18.2%)	2 (40%)	0
No strong preference	3 (27.3%)	0 (0%)	3 (50%)
*Ethical issues when asking children to answer the valuation tasks?*			
No any ethical issue	8 (46.7%)	4 (44.4%)	4 (66.7%)
The "death" issue	7 (46.7%)	5 (55.6%)	2 (33.3%)

^*^Only participants preferred DCE (*n* = 11) provided the responses of this question

### EQ-5D-Y-3L versus EQ-5D-Y-5L

As shown in Table 2, most of the participants (*n* = 11, 73.3%) preferred the EQ-5D-Y-5L to the EQ-5D-3L. They considered that the EQ-5D-Y-5L provides more response options and thus increases the discriminatory ability to detect changes in health status. Three participants (20.0%) preferred using the EQ-5D-Y-3L with the concern that the five-level version may increase the difficulty of distinguishing between response options by children. One participant (6.7%) had no preference between the two versions as both had advantages and disadvantages.

### Source of health state preferences

Twelve (80.0%) participants suggested that children should be included in child health valuation (Table 2). They thought that children can provide preferences from their unique perspectives that adults may not be able to. They disagreed with the rationale of using the adult’s preferences because they can vote or pay taxes. “*I think adolescents are members of the public. When talking about allocation of the budget or investments in their health and health care programs for children, I think their views matter, even if they’re not taxpayers.*” [*H*1], and “*if you’re asking someone to value a health state, it should be people who are actually users of the healthcare system instead of taxpayers.*” [*A*3]. “*I think [this recommendation] is a highly problematic set of assumptions in terms of understanding what our society values and is worth willing to pay for. The extension of that logic is, if you pay more tax, you have more say, so that wealthier people in a progressive taxation system should have more say in terms of which things are valuable. Or if you don’t have a say, because you have cognitive difficulties, or you’re unemployed, or what have you, that you have less say*.” [*H*2].

In contrast, two participants (13.3%) supported using adults as the source of preferences because that is the societal preferences recommended in Canada HTA guidelines. Moreover, they argued that there is no separate budget for children and adolescents. One participant suggested using a mix of both adults and children since they are all part of society.

When asked about cognitive capacity of children, most participants thought that older adolescents should be able to comprehend and answer questions related to health state preferences. In general, they recommended approaching adolescents aged 12 years or older.

Health state preference tasks may ask for trade-off of life years for quality of life or compare health states with immediate death. There may be potential ethical concerns when asking children to complete these questions. The view on the ethical issue was split among the participants. About half (*n* = 8, 53.3%) deemed that there was no ethical concern. They mentioned that although the experience likely varies depending on individual experience, death was not a foreign subject for children. “*Children are pretty aware of dying and illness and disability through many sources of information, e.g., the social media, nowadays*.” [*G*1], and “*Children know what they’re going to be talking about, and knowing that it’s theoretical, and it doesn’t really mean it’s going to happen.*” [*A*5]. In contrast, 7 (46.7%) participants thought that we should be cautious when talking about “death” with children. “*I think that does create a risk and potential sort of triggering of fears.*” [*A*2], “*I think it’s a really important one to consider. They’ve experienced death of pets and older family members but they don’t fully understand it the way an adult does.*” [*G*2], and “*You would have to get permission from their parents or guardian first.*” [*I*2].

### Perspective

Among the 12 participants who suggested using children’s preferences, they all suggested that the valuation tasks be framed from the child’s own perspective. One of the two participants who suggested using adults as the source of preference recommended the perspective of own child’s friend or peer group, while the other suggested the perspective of a general child without any specification (Table 2).

### Preference elicitation methods

As illustrated in Table 2, most of the participants (n = 11, 73.3%) preferred DCE to TTO. They considered that DCE was straightforward, easier, and closer to reality than TTO. Among them, six preferred DCE with duration to DCE with death. They thought that the DCE with duration was more detailed and realistic than DCE with death. “*I think kids understand if it’s a question of how long I’m going to live, and it is easier for them to wrap their heads around, or safer from an ethics perspective.*” [*A*2], while “*In DCE with death, the concept of immediate death is very romantic. If I can’t have what I want, I’d rather be dead. While DCE with duration might be better because there is no option to go with the highly romantic thing that might not reflect their true preference.*” [*H*4]. Two participants preferred DCE with death and viewed that adding the duration dimension into the choice set could increase the cognitive burden.

Two participants (13.3%) preferred TTO. They deemed that TTO tasks were more intuitive than DCE tasks in which it is not easier to differentiate between two choices. “*You need that concrete, like the immediate death, to provide a concrete idea for a kid in their mind*” [*G*3]. The remaining two participants (13.3%) had no strong preferences between the two methods.

As demonstrated in Table 2, there were some divergences in choosing preference elicitation methods between the participants with prior experience of utility measurement and those without. For example, all six participants without the prior experience preferred the DCE approach and four of them (66.7%) considered no ethical issues when directly asking children to answer the valuation tasks.

## Discussion

Measuring and valuing child health poses unique methodological and normative questions. Stakeholder engagement can provide a useful insight to tackle these issues. Canadian stakeholders suggested that we elicit health state preferences from children and adolescents directly from their own perspective. Furthermore, most participants considered DCE with duration an easier preference elicitation method compared with other types of DCE or TTO. This stakeholder engagement provides important feedback that will inform the design of child health valuation in Canada.

Assessing innovative health technologies for pediatric populations requires patient-reported outcome measures suitable for children. There are 15 preference-based instruments that have already been developed and validated to measure health for children of various ages [38, 39]. These instruments were designed to generate health utilities. Empirical studies have consistently found that children’s health state preferences were different from those of adults [4, 5]. This was a key rationale for a separate value set for the EQ-5D-Y stated in the international protocol [16]. However, when it comes to the source of preferences, the protocol recommends using general adult populations based primarily on the legal age threshold for voting and paying taxes (18 years in most countries) [16]. Most of our stakeholders disputed the legal age justification for child health valuation. They provided convincing arguments that the voices of cognitively able children should be considered when allocating healthcare resources. Only two participants in our interviews who supported the use of adults (both happened to be health economists) stated that the use of adult preferences would conform to the current HTA guidelines. However, it is important to note that current guidelines are for adults and in fact no HTA agencies have provided any recommendations on child health valuation [38]. If a separate value set for a child preference-based instrument is needed because health state preferences differ between children and adults, why are the preferences of children not used in developing the value set?

Ethical concerns were another factor for the recommendation in the protocol. It is true that talking about death could be sensitive and have potential impact on some children. However, it might not be possible to completely shield children from being exposed to these subjects in the internet era. Some participants raised the concern, but they nonetheless considered that children should be asked for health state preferences if we approach this carefully and with the consent from parents. Indeed, there are alternative designs to avoid talking about death in the valuation task, for instance, DCE with duration.

A complication of recommending the use of preferences from adults as proxy for child health valuation is from which perspective the tasks should be framed. Adults are asked to state their preferences from the perspective of a 10-year-old child according to the protocol [16]. In general, many adults (parents in particular) would agree that it is difficult to know what a child thinks or prefers. Moreover, the 10-year-old child perspective was an arbitrary choice initially [4], but subsequently has been used by other studies for comparability [5, 10, 14]. This perspective, or any perspective other than the respondent’s own, creates new challenges. Evidence has shown that the image of a hypothetical 10-year-old child is different among adults, which caused variations in health state preferences [8, 40]. Furthermore, adults’ preferences change with the age of the hypothetical child [41]. Using the child’s own perspective will avoid this complication.

Given the challenges in child health valuation, an international protocol can provide methodological guidance and allow for comparability across studies. However, addressing some of these challenges requires empirical evidence, for others they impose normative questions that go beyond science. Therefore, stakeholder engagement can be important to inform the design of child health valuation to align with the expectations of local stakeholders [30]. Unfortunately, it is rare that stakeholders are engaged prior to a child health valuation study for various reasons. The recently published roundtable discussion in the US was one of the few [42]. Most of US stakeholders expressed that adolescents could relate to a 10-year-old’s perspective better than adults and were capable of self-completing valuation tasks, and thus should be directly included in the valuation study. They also raised concerns that adults would be inconsistent in their views about a 10-year-old [42]. Similar to what we found in our study, US stakeholders who were more familiar with HTA were also more inclined to favor the use of adult preferences based on the legal age for voting and paying taxes [42].

There are a few strengths in our stakeholder engagement. First, interviews were chosen to allow us to have sufficient time to engage with each participant and obtain their insights into this rather complex topic. We preferred interview to other qualitative study designs (e.g., focus group) so we can capture the views from individual stakeholders with diverse background and expertise, instead of reaching a consensus among them. Second, our stakeholders include pediatricians, child health researchers, and a bioethicist who can share their direct experience with children. Another strength is that all interviews were conducted by one author, which ensured consistency across the interviews.

One limitation of this qualitative study was that we only recruited those who were highly educated health care professionals or experts in health-related research. We were concerned about the complexity of this topic for laypersons in the public. The two participants who were not familiar with the concept of health utility felt that some parts of the interview (e.g., preference elicitation methods) were rather technical. During the interview, we used examples of TTO and DCE tasks to help participants understand the main process of preference elicitation. Providing introductive material in advance may be also helpful to facilitate understanding. Another limitation is that we designed the interview by focusing on the issues around child health valuation methods. Their potential impact on health policy making was only briefly discussed due to the time constraint. We felt this issue is nonetheless important, especially for decision makers, to be aware of important implications related to child health valuation.

## Conclusions

Most Canadian stakeholders supported eliciting health state preferences directly from adolescents using their own perspective for child health valuation. DCE was considered a more appropriate preference elicitation method for this age group. Future studies should assess the feasibility of directly eliciting preferences for the EQ-5D-Y from adolescents in Canada.

### Supplementary Information

Below is the link to the electronic supplementary material.

Supplementary file1 (PDF 177 kb)

Supplementary file2 (PDF 360 kb)

## Data Availability

Not applicable.
