# Supplementary material for: Understanding Canadian stakeholders’ views on measuring and valuing health for children and adolescents: a qualitative study
Source: Qual Life Res. 2024 Mar 5;33(5):1415–22. doi: 10.1007/s11136-024-03618-y (PMC11045599; doi:10.1007/s11136-024-03618-y)
Supplement: Supplementary file 1 — Supplementary file1 (PDF 177 kb) [file 11136_2024_3618_MOESM1_ESM.pdf]

Interview Guide for

Understanding the views of Canadians on valuing health for children and adolescents

Version 2.0

INTERVIEWER CHECKLIST

- Make sure a Zoom meeting room has been set up before the interview
- Share the meeting link with the participant in advance
- Remind the participant one day before the meeting
- Email the consent form to the interviewee in advance
- Set up an audio recorder and test the zoom recording feature as well

Script 1: Verbal Consent

{Note to Interviewer: verbal consent must be obtained before proceeding with the interview}

Hello, thank you for taking time to talk to me today. I am Feng Xie, Professor of Health Economics at McMaster University.

A consent form was emailed to you. The form provides the details about the study. Before beginning the study, I would like to go through it with you.

{Note to Interviewer: Share Zoom screen with the Consent Form open}

You are being invited to take part in a research study that will help improve our understanding of Canadians' view on valuing health for children and adolescents. Your agreement to participate in this study is optional and entirely up to you.

If you choose to participate in this study, we will start by having you answer a few questions to provide us with some background information about you (for example, age and education), your current position, prior experience in health economics in general, and any experience or knowledge in health utility measures. We will then proceed with an interview aimed at seeking your view on how to value youth health to support reimbursement or coverage policy making in Canada. There are no physical risks to participating in this study. This interview will take between 45 and 60 minutes of your time. You will not incur any expenses as part of this study. At the same time, we will provide a Tim Horton coupon of $50 as a token of appreciation for your time of participation.

It's important to note that this study is using the McMaster Zoom platform to conduct the interview, which is an externally hosted cloud-based service. A link to their privacy policy is available on the consent form that was emailed to you. Please note that while this service is approved for collecting data in this study, there is a small risk with any online platform such as this that is collected on external servers falling outside the control of the research team. Please let us know if you have any concerns. Our contact information is listed on the consent form.

In terms of benefits of participation, you will not receive any direct benefit from participating in this study. However, your participation in this study will help us understand Canadians' views on valuing youth health and provide better support to health policy making in Canada.

Your participation in this study is voluntary. You may decide not to participate in this interview, or withdraw from it at any time during the interview. You can refuse to answer any question that you don't feel comfortable to answer.

The interview will be recorded and transcribed verbatim for analysis. All personal information that could be used to identify you will be de-identified in the analysis. We plan to publish this study in peer-reviewed academic journals, but personal information will not appear in any publications or presentations that come out of this study.

All information collected from this study will be kept in a password-protected computer for 10 years. Only the study team has the access to the study records. Representatives of the Research Ethics Board

may access the study records and your personal information to check that the information collected for the study is correct and to make sure that this study followed proper laws and guidelines.

Do you have any questions?

{Note to interviewer: Answer all questions before proceeding}

Do you agree to participate in this study knowing that you can withdraw at any point with no consequences to you?

While we require your verbal consent to begin the study, we do ask that you sign the consent form that was emailed to you and return it via email for our records.

{If yes, record verbal consent and proceed with the interview, If no, thank the participant for their time and end the Zoom call}

Script 2: Introduction

{Note to interviewer: the following introduction may be SHORTENED for interviewees who are health economists and familiar with Canada's regulatory and coverage approval process}

A new health technology, a drug or medical device, needs to receive regulatory approvals before it can be used on patients. First, the safety and efficacy of the technology needs to be approved by Health Canada so it can be sold in Canada. Second, the technology needs to be comprehensively assessed through a process called health technology assessment (HTA) by a quasi-government agency known as the Canadian Agency for Drugs and Technologies in Health (CADTH). Only those technologies that are safe, effective, cost-effective, and affordable may be covered by the Canadian publicly funded health care systems. A key component of HTA is to compare the cost-effectiveness of the new technology with existing technologies for treating the same disease.

A recommended way of evaluating cost-effectiveness is to compare cost and health benefits of patients who use the technology whereby the health benefit is assessed by weighing survival with quality of life, known as quality-adjusted life years (QALYs). Quality of life is usually measured using an instrument that has been developed and tested. Patients are asked to answer all questions contained in that instrument according to their health status.

*{**Note to interviewer: showing the EQ-5D-3L slide to the interviewee**}*

The EQ-5D 3 level version (EQ-5D-3L) was one of such instruments that was developed in 1990s. It has five questions related to mobility, self-care, usual activities, pain/discomfort, and anxiety/depression. For each question, there are 3 response options: no problems, some problems, extreme problems. The respondent is asked to answer each question by ticking one of the three options in each question. For example, if a patient chose no problems in mobility and self-care, some problems in usual activities, moderate pain/discomfort, and severely anxious and depressed. This describes this person's health status. We can also use a five-digit number to represent it, 11223.

This type of instruments comes with a formula that assigns a single index number with 1 representing perfect or full health and 0 for dead to every *health status described by the instrument*. Using the previous example, let us take a look at how this formula works.

*{**Note to interviewer: showing and explaining the EQ-5D-3L UK Algorithm slide to the interviewee**}*

To develop such a formula, a representative sample of *adults* from the general public is surveyed to obtain their preferences about selected health status. This is known as health state valuation.

*{**Note to interviewer: showing the EQ-5D-Y-3L slides to the interviewee**}*

The EQ-5D-Y, a child-friendly version of the EQ-5D, has been developed in 2010. This child version is different in some of the wordings appropriate for children and adolescents.

*{**Note to interviewer: showing the comparison between Y-3L and Y-5L slide to the interviewee**}*

In the meantime, the EQ-5D-Y-5L, a revised version of EQ-5D-Y with expanded response levels, has been developed to measure health for children and adolescents with improved sensitivity. Which one do you prefer and why?

*{**Note to interviewer: showing the TTO slide to the interviewee**}*

For the EQ-5D, two methods have been recommended to conduct health state valuation study. One is called time trade-off (TTO in short) and the other discrete choice experiment (DCE in short). I will briefly go over both methods.

TTO involves a trade-off between quality of life and quantity of life. It usually presents the question in a timeframe of 10 years. Under a hypothetical scenario, out of 10 years, how many life years a person is willing to give up in order to avoid living in an impaired health state?

For example, on this slide, there are two options, Life A and Life B. Life A is living in full health for 10 years and followed by death. The number of years in Life A can be changed. Life B is living in a health state described by the EQ-5D-3L *{**note to interviewer, read the health states on the slide**}*, with a fixed 10 years followed by death. The respondent is asked a question "what do you prefer, Life A, Life B, or Life A and Life B are about the same?". Because the health state in Life B with a few impaired domains, and thus worse than full health in Life A, it would be reasonable to expect that most of interviewers, if they understand the question, would choose Life A.

*{**Note to interviewer: showing the TTO slide to the interviewee**}*

Then, the number of year of Life A will be changed to 5 years. If the respondent said that the two options are about the same. We can then calculate the utility as 5/10 =0.5. Then move on to next state.

If the respondent prefers Life A, it means the respondent is willing to give up 5 years of his/her life to avoid being in Life B for 10 years. Then the number of years in Life A will decrease to 4 years. We continue until the respondent indicates the two are about the same.

Or if the respondent prefers Life B, it means the respondent is NOT willing to give up 5 years of his/her life to avoid being in Life B for 10 years. Then the number of years in Life A will increase to 6 years. So on and so forth, until the respondent indicates the two are about the same.

As you can see, TTO questions go back and forth and so are quite complex and time consuming. Even in a hypothetical situation, not everyone is comfortable if talking about death or trading off life years. Other alternative way has been used.

*{**Note to interviewer: showing the DCE slide to the interviewee**}*

This is a method called Discrete Choice Experiment, DCE in short. It presents two health states and ask the respondent to choose which one they prefer. That is all in DCE questions. Sometimes, both TTO and DCE are used in combination.

In both TTO and DCE, since all respondents are adults, we ask them to tell us their preferences by imaging living in those health states. Now let us move to a child-friendly version of the EQ-5D that is used for children between 8 and 15 years.

Now we want to value the health states for the child version as we did for the adult version. But we have a few challenges in the design for which we want to seek your view today.

Script 3: Questions on perspective

For the adult version, respondents are adults recruited from the general public. Reasons for choosing the general public include a) they are the taxpayers to support the public health care systems; and b) they understand the questions; and c) they don't have lived experience with impaired health state so can provide impartial responses.

For the youth version, WHO do you think should be asked to complete these valuation tasks, adults, adolescents who are old enough to understand and answer the questions, or someone else?

*{**Note to interviewer: probing the interviewee with stating that evidence shows that adults' valuations for children are consistently higher than for themselves***

***Other probing questions: arguments against the use of adolescents are they may not understand the questions; they are not old enough to pay tax or vote yet; there might be ethical concerns when talking about death with them**}*

Script 4: Questions on framing

Another complexity in the design is how to ask the questions, we call it framing. There are a few alternative ways: considering yourself as child, your own child (parents only), another child you know, or a non-specific child.

What is your view on this?

Would different framings affect your answers?

Do you think a further description, for example, age, of a hypothetical child may help answer the valuation question or should the description be general?

{Note to interviewer: probing the interviewee by asking whether s/he has any suggestion on how to describe the hypothetical child}

Script 5: Questions on TTO and DCE

Now I will ask you a few questions about comparing between two methods that have been used to measure preferences, TTO and DCE.

*{**Note to interviewer: showing the TTO and DCE slides to the interviewee**}*

First of all, let us have a review on the TTO and DCE questions.

Do you think valuing health state for a child is feasible for both methods?

*{**Note to interviewer: showing the DCE slide to the interviewee**}*

In DCE, there is no back-and-forth in varying the number of years. However, the DCE responses alone are not sufficient. We have two options, adding a few TTO questions or modifying the current DCE design by, for example, adding duration of each choice option or adding a third option of "dead".

*{**Note to interviewer: showing the DCE with additional information slide to the interviewee**}*

What do you think about these two different approaches? This is a rather technical question. Let me know if you are not comfortable or don't know how to answer them.

Do you think there are any ethical concerns when children and adolescents answering these valuation questions?

Script 6: Potential implications on coverage policy making in Canada

The different design for the child version may lead to the different values which subsequently could affect cost effectiveness results compared with the adult version. This may have implication on decisions on covering new treatments for children and adolescents vs adults.

Do you think the child version should have its own value set or use the value set from the adult version? I would like you to think about this question and answer it while keeping in mind the policy implication I just mentioned.

Do you have any other comment or question?

If no, thank you very much for your participation and feedback.

{Note to the interviewer: Stop the recording and the meeting platform}

END OF INTERVIEW
